# The association between burnout and empathy in intern medical doctors rotating at Steve Biko Academic Hospital in Pretoria, South Africa

**DOI:** 10.4102/jcmsa.v4i1.323

**Published:** 2026-07-08

**Authors:** Quanita N. Tatchell, Melissa Fernihough, Gian Lippi

**Affiliations:** 1 Department of Psychiatry, Faculty of Health Sciences, University of Pretoria, Pretoria, South Africa

**Keywords:** burnout, empathy, internship, medical doctors, occupational stress, mental health, Maslach Burnout Inventory, Jefferson Scale of Empathy

## Abstract

**Background:**

Burnout, a state of mental and physical exhaustion from chronic work stress, affects approximately one in three doctors. Burnout is associated with substance use, suicidality and impaired doctor-patient relationships. It also contributes to reduced quality of care, increased medical errors and malpractice claims. Empathy is the cognitive ability to understand and respond to a patient’s experiences. This study explored the relationship between burnout and empathy among South African intern doctors.

**Methods:**

This cross-sectional quantitative study used consecutive sampling. Self-administered questionnaires were completed by 95 interns. Data were analysed using descriptive statistics, as well as bivariate and multivariable analyses.

**Results:**

The median age of interns was 26 years. Over 70% were overextended or burnt out. About one-third (33.68%) of participants had a diagnosed mental illness, and over half reported worsening illness during their internship. Lack of awareness of counselling services (83.16%) was associated with higher burnout (*p* = 0.020). Age was associated with burnout profile (*p* < 0.001), with younger interns more likely to be burnt out. Empathy scores were significantly lower in the burnt out, overextended and ineffective interns (*p* = 0.002), even after adjustment for demographic, clinical and work-related factors.

**Conclusion:**

The high prevalence of burnout among intern doctors and its inverse association with empathy underscore the need for systemic, evidence-based interventions to reduce burnout and enhance empathy, supporting the delivery of quality patient care.

**Contribution:**

This study offers novel insights into the challenges faced by South African intern doctors, providing a foundation for future research and targeted institutional reform.

## Introduction

The practice of medicine can be meaningful and fulfilling, but it is also often accompanied by high demands and chronic stress. Globally, approximately one in three medical doctors experiences burnout at any given time.^1^ The term burnout was coined in 1974 by Freudenberger, who defined it as ‘a state of mental and physical exhaustion caused by one’s professional life’.^2^ This state of exhaustion undermines physicians’ well-being, contributing to strained personal relationships, substance use disorders and suicidality.^3,4^ Professionally, burnout adversely impacts doctor-patient relationships, reducing patient satisfaction, leading to poorer quality of care and an increased risk of medical errors.^3,5^ These consequences may further drive institutional challenges such as malpractice claims and resource strain, perpetuating a cycle of stress and exhaustion within healthcare systems.^3,5^ The coronavirus disease 2019 (COVID-19) pandemic further intensified these pressures, with heightened workloads and emotional demands contributing to increased levels of burnout across the medical fraternity.^5^

Burnout is of particular concern in the South African healthcare setting, where limited resources, long working hours, compulsory overtime and restricted leave exacerbate stress among doctors.^6,7,8,9,10^ Studies have reported high rates of burnout across the continuum of medical training and early clinical practice in South Africa, including among medical students, community service doctors, medical officers and registrars.^4,6,7,9,10,11^ However, little is known about burnout during the internship period, a compulsory 2-year rotation through various medical disciplines undertaken by newly qualified doctors. This transitional stage bridges undergraduate training and independent clinical practice; burnout during internship may therefore have long-term implications for professional development and patient care.^5,10,12^

Empathy is closely linked to the practice of medicine and may both influence and be influenced by burnout.^13,14^ Empathy has been described as a predominantly cognitive attribute involving understanding patients’ experiences, concerns and perspectives, coupled with the ability to communicate this understanding and the intention to help.^14,15,16^ It encompasses cognitive empathy (understanding the emotional state of the patient) and affective empathy (experiencing an emotional response to the feelings of others).^13,14,15,16^ Empathy is central to building trust, enhancing patient satisfaction and improving clinical outcomes.^15,16^ For example, studies showed that physicians who had higher empathy scores, as measured by the Jefferson Scale of Empathy-Health Professions (JSE-HP), achieved better control of chronic conditions such as diabetes and had patients with fewer complications and hospitalisations.^15,16^ Similarly, medical students with higher empathy scores were rated higher for their clinical competence.^15,16^

The relationship between burnout and empathy has been the subject of considerable debate.^13,14^ They have been increasingly recognised as interconnected constructs that may influence one another in complex ways.^13,14^

Some studies suggest a negative association between burnout and empathy, arguing that empathy may buffer against stress by improving patient outcomes, whereas burnout may diminish the capacity for empathy.^13,14^ Conversely, existing burnout may impair the development of therapeutic relationships, lower tolerance and diminish clinicians’ capacity for empathy.^13,14^ Other studies propose that high empathy could predispose healthcare professionals to burnout because of emotional strain and repeated exposure to suffering.^13,14^ Over time, empathy scores may decline as an adaptive mechanism to reduce emotional overload and mitigate burnout.^7,13^

To our knowledge, no studies have investigated the association between burnout and empathy among South African intern doctors using validated measures such as the Maslach Burnout Inventory-Human Services Survey for Medical Personnel (MBI-HSS [MP]) and the JSE-HP. Addressing this gap is critical, as understanding how burnout and empathy are linked in this population may inform strategies to foster resilience, preserve empathy and safeguard quality healthcare delivery.

This study aimed to determine the presence and degree of burnout and empathy among South African medical interns and to explore associations between the two.

## Research methods and design

This quantitative, observational, exploratory cross-sectional study was designed to explore the relationship between burnout and empathy among South African medical interns.

The study was conducted at Steve Biko Academic Hospital (SBAH), a public-sector tertiary teaching hospital in Pretoria, South Africa, affiliated with the University of Pretoria. Medical interns rotate through various specialist departments at SBAH as part of their compulsory 2-year internship programme.

### Sampling

The study population included all first- and second-year intern doctors employed at SBAH during the study period (*N* = 128). Of these, 97 interns participated (response rate: ~76%), with 95 providing fully completed questionnaires (completion rate: ~74%).

A non-probability, consecutive sampling strategy was used. This was done to have representation across all interns’ departmental rotations.

### Data collection

Data were collected using self-administered paper questionnaires between 01 December 2023 and 29 February 2024.

Sessions were scheduled before or after departmental academic meetings to minimise disruptions to clinical duties and optimise access to as many interns as possible. Intern group leaders facilitated meetings in which the principal investigator met with participants from each department.

The principal investigator explained the study, obtained informed consent and distributed the questionnaires. Group leaders facilitated distribution to interns unable to attend sessions, with the principal investigator following up for collection.

The questionnaire included demographic items, potential confounders and two validated scales. Burnout was measured using the MBI-HSS (MP) scale, and empathy was measured using the JSE-HP.^15,17,18^

The MBI-HSS (MP) is a 22-item instrument comprising three subscales: emotional exhaustion (EE), depersonalisation (DP) and personal accomplishment (PA), as outlined in Table 1.^9^

**TABLE 1:** Subscales of the Maslach Burnout Inventory-Human Services Survey for Medical Personnel.

Subscale	Description	Low	Moderate	High
Emotional exhaustion	Feeling emotionally depleted and lacking energy to face another workday	≤ 16	17–26	≥ 27
Depersonalisation	Development of negative, detached attitudes toward work or people	≤ 6	7–12	≥ 13
Personal accomplishment	A sense of competence and achievement in one’s work	≤ 31	32–38	≥ 39

*Source*: Sirsawy U, Steinberg W, Raubenheimer J. Levels of burnout among registrars and medical officers working at Bloemfontein public healthcare facilities in 2013. S Afr Fam Pract. 2016;1(1):1–6. https://doi.org/10.4102/safp.v58i6.4610

Previous South African studies have commonly applied categorical cut-off ranges to classify burnout severity based on the MBI-HSS (MP) subscales.^6,7^ The fourth edition of the MBI manual places greater emphasis on profile categories.^19^ Both approaches are consistent with the conceptualisation of burnout as a multidimensional construct.

Average scores for EE, DP and PA were used to classify participants into burnout profile groups based on the MBI framework, as shown in Table 2.^20,21^

**TABLE 2:** Burnout profile classification criteria.

Burnout profile	Description	Mean score		
		EE cut-off	DP cut-off	PA cut-off
Engaged	Emotionally connected to their work, productive and not experiencing burnout	≤ 3	≤ 2.73	> 4.66
Ineffective	Perceive themselves as underperforming or less competent in their roles	≤ 3	≤ 2.73	≤ 4.66
Overextended	Feel overburdened or stressed but have not yet reached full burnout	> 3	≤ 2.73	≤ 4.66
Burnout	Exhibit high emotional exhaustion, detachment from work and feelings of ineffectiveness	> 3	> 2.73	≤ 4.66

*Source*: Archer E, Turner R. Measuring empathy in a group of South African undergraduate medical students using the student version of the Jefferson Scale of Empathy. Afr J Prim Health Care Fam Med. 2019;11(1):e1–e5. https://doi.org/10.4102/phcfm.v11i1.1956; Dyrbye LN, Shanafelt TD, Sinsky CA, et al. Burnout among health care professionals: A call to explore and address this underrecognized threat to safe, high-quality care. NAM Perspect. 2017;7(7):1–11. https://doi.org/10.31478/201707b

EE, emotional exhaustion; DP, depersonalisation; PA, personal accomplishment.

Empathy was assessed using the JSE-HP, which is a 20-item tool with reversed-scored items (1, 3, 6, 7, 8, 11, 12, 14, 18 and 19 on a Likert scale [Strongly agree = 1 to strongly disagree = 7]) and directly scored items (Strongly disagree = 1 to strongly agree = 7). Higher total scores reflected greater empathic orientation.^20^

### Data analysis

Data were captured in Microsoft Excel and analysed in SAS Version 9.4. Descriptive statistics were used to summarise the data. Categorical variables were presented as frequencies and percentages, while continuous variables were summarised as means with standard deviations or medians with percentiles, depending on distribution.

The Shapiro–Wilk test was used to assess normality. Chi-square or Fisher’s exact tests were used for categorical comparisons and Mann–Whitney *U* or Kruskal–Wallis tests for continuous variables. Correlation analyses were conducted to explore associations between burnout and empathy. A significance level of *p* < 0.05 was applied.

Burnout was analysed using both continuous and categorical approaches. Spearman’s rank correlation coefficients were calculated to assess associations between continuous burnout subscale scores and empathy scores. For categorical analyses, burnout profiles were derived from MBI subscale scores using established interpretive guidelines.

Associations between burnout profiles and categorical variables were assessed using chi-square or Fisher’s exact tests, while comparisons of continuous variables, including empathy scores, across burnout profiles were performed using the Mann–Whitney *U* or Kruskal–Wallis tests.

Given the exploratory nature of the study and the sample size, analyses focused primarily on descriptive statistics and bivariate associations. Multivariable modelling was conducted to further explore the relationship between burnout and empathy while adjusting for selected variables, including age, gender, medical school attended, overtime working hours, mental health history and awareness of counselling services; however, these findings are interpreted as exploratory rather than indicative of independent predictors.

### Ethical considerations

Ethical clearance to conduct this study was obtained from the Faculty of Health Sciences Research Ethics Committee, University of Pretoria (No. 553/2023).

## Results

### Participant characteristics

Ninety-seven interns participated in the study. Two did not complete the questionnaires; thus, 95 were included in the final analysis. The median age was 26 years (interquartile range [IQR]: 25–29 years), and 66.32% were female. The majority (83.16%) did not hold a degree prior to attending medical school.

A slightly higher proportion of participants were in their second year of internship (53.68%). Most participants (57.89%) reported working > 40 hours but < 80 hours of overtime per month. The University of Pretoria was the most frequently represented training institution (38.95%). Just over half of participants (51.58%) felt unprepared by their university to cope with stress and work–life balance.

Approximately one-third (33.68%) of interns reported a diagnosed mental illness, 56.25% of whom experienced worsening symptoms during internship.

**FIGURE 1:**
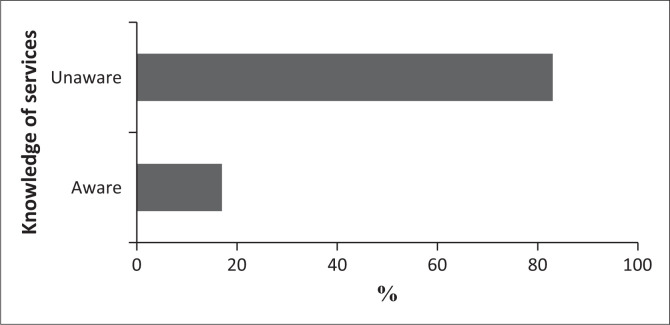
Interns’ awareness of counselling services.

Most interns were unaware of hospital counselling services (83.16%), as shown in Figure 1. The majority of participants (93.68%) reported receiving emotional support from family or friends. Detailed participant characteristics are presented in Table 3.

**TABLE 3:** Participant characteristics (*N* = 95).

Characteristics	*n*	%	Median	IQR
**Demographics**				
Age (years)†	-	-	26	25–29
Female	63	66.32	-	-
Male	32	33.68	-	-
**Relationship status**				
Committed relationship	41	43.16	-	-
Single	37	38.95	-	-
Married	15	15.79	-	-
Divorced	1	1.05	-	-
Other (it’s complicated)	1	1.05	-	-
**Education and training background**				
**Previous degree or qualification**				
No	79	83.16	-	-
Yes	16	16.84	-	-
**Medical training institution**				
University of Pretoria	37	38.95	-	-
Sefako Makgatho Health Sciences University	15	15.79	-	-
University of Cape Town	7	7.37	-	-
University of the Witwatersrand	8	8.42	-	-
University of the Free State	8	8.42	-	-
University of KwaZulu-Natal	3	3.16	-	-
Stellenbosch University	2	2.11	-	-
University of Limpopo	1	1.05	-	-
University in Cuba	8	8.42	-	-
University in China	4	4.21	-	-
Internationally trained: Other	1	1.05	-	-
Other	1	1.05	-	-
**Institutional preparation for internship**				
Felt unprepared	49	51.58	-	-
Felt adequately prepared	46	48.42	-	-
**Work-related**				
**Internship year**				
First year	44	46.32	-	-
Second year	51	53.68	-	-
**Current departmental rotation**				
Family medicine	25	26.32	-	-
Internal medicine	14	14.74	-	-
General surgery	11	11.58	-	-
Paediatrics	10	10.53	-	-
Psychiatry	10	10.53	-	-
Obstetrics and gynaecology	9	9.47	-	-
Orthopaedics	9	9.47	-	-
Anaesthesiology	7	7.37	-	-
**Overtime hours per month**				
≤ 40	4	4.21	-	-
> 40 but < 80	55	57.89	-	-
≥ 80	36	37.89	-	-
**History of diagnosed mental illness**				
No	63	66.32	-	-
Yes	32	33.68	-	-
**Diagnosed mental illness**				
Major depressive disorder (MDD)	18	56.25	-	-
Any anxiety disorder (AD)	14	43.75	-	-
Bipolar disorder (BD)	2	6.25	-	-
Schizophrenia	0	0.00	-	-
Any personality disorder (PD)	1	3.13	-	-
Obsessive compulsive disorder (OCD)	2	6.25	-	-
Other-not specified	3	9.38	-	-
**Co-occurring diagnoses**				
MDD and AD	6	18.75	-	-
MDD and PD	1	3.13	-	-
MDD and OCD	1	3.13	-	-
AD and BD	1	3.13	-	-
**Timing of diagnosis**				
Before internship	25	78.13	-	-
During internship	7	21.88	-	-
**Worsening of mental illness during internship**				
Yes	18	56.25	-	-
No	14	43.75	-	-
**Support and resources**				
**Awareness of counselling services available at SBAH**				
Unaware of hospital counselling services	79	83.16	-	-
Aware of counselling services	16	16.84	-	-
**Family geographical proximity**				
Pretoria	40	42.11	-	-
Gauteng	29	30.53	-	-
Family elsewhere in South Africa	25	26.32	-	-
Family in another country	1	1.05	-	-
**Perceived emotional support from family or friends**				
Not receiving emotional support	6	6.32	-	-
Receiving emotional support	89	93.68	-	-
**Frequency of emotional support received**				
Daily	40	44.94	-	-
Weekly	30	33.71	-	-
Monthly	15	16.85	-	-
Few times a year	4	4.49	-	-

IQR, interquartile range; SBAH, Steve Biko Academic Hospital.

† , Age range: 23–45 years.

### Burnout

**FIGURE 2:**
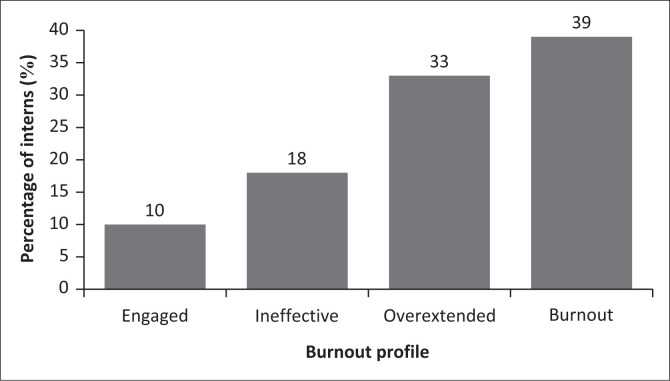
Interns’ burnout profiles.

Based on MBI scores, interns were classified into four burnout profiles: engaged (10.53%), ineffective (17.89%), overextended (32.63%) and burnout (38.95%), as shown in Figure 2.

### Factors associated with burnout

Age was significantly associated with burnout profile (*χ*^2^(3) = 19.31, *p* < 0.001), with interns in the engaged profile having a higher median age (29.50 years) than those in the burnout profile (26.00 years).

The medical school attended was significantly associated with burnout profile (*χ*^2^(33) = 71.30, *p* < 0.001), with higher burnout rates observed among graduates of the University of Pretoria (59.46%), the University of Cape Town (57.14%) and Sefako Makgatho Health Sciences University (40.00%).

Awareness of counselling services available at SBAH showed a significant association with burnout profile (Fisher’s exact test, *p* = 0.020), with lower proportions of burnout observed among interns who were aware of available services.

No significant associations were observed for gender, relationship status, prior degree, perceived institutional preparation, internship year, rotation, overtime hours, history of mental illness, timing of diagnosis, worsening of mental illness or social support variables (Table 4).

**TABLE 4:** Associations between study variables and burnout profile (*N* = 95).

Variables	Overall				Engaged				Ineffective				Overextended				Burnout				*χ* ^2^	*df*	*p*-value
	*n*	%	Median	IQR	*n*	%	Median	IQR	*n*	%	Median	IQR	*n*	%	Median	IQR	*n*	%	Median	IQR			
**Demographics**	-	-	-	-	-	-	-	-	-	-	-	-	-	-	-	-	-	-	-	-	-	-	-
Age (years)†	-	-	26	25–29	10	-	29.50	28–30	17	-	26	25–28	31	-	27	25–29	37	-	26	25–26	19.31	3	< 0.001**
**Gender**	-	-	-	-	-	-	-	-	-	-	-	-	-	-	-	-	-	-	-	-	3.53	3	0.318
Female	63	66.32	-	-	5	7.94	-	-	14	22.22	-	-	19	30.16	-	-	25	39.68	-	-	-	-	-
Male	32	33.68	-	-	5	15.63	-	-	3	9.38	-	-	12	37.50	-	-	12	37.50	-	-	-	-	-
**Relationship status**	-	-	-	-	-	-	-	-	-	-	-	-	-	-	-	-	-	-	-	-	-	-	0.204††
Committed relationship	41	43.16	-	-	6	14.63	-	-	8	19.51	-	-	14	34.15	-	-	13	31.71	-	-	-	-	-
Single	37	38.95	-	-	3	8.11	-	-	5	13.51	-	-	14	37.84	-	-	15	40.54	-	-	-	-	-
Married	15	15.79	-	-	0	0.00	-	-	3	20.00	-	-	3	20.00	-	-	9	60.00	-	-	-	-	-
Divorced	1	1.05	-	-	1	100.00	-	-	0	0.00	-	-	0	0.00	-	-	0	0.00	-	-	-	-	-
Other (it’s complicated)	1	1.05	-	-	0	0.00	-	-	1	100.00	-	-	0	0.00	-	-	0	0.00	-	-	-	-	-
**Education and training background**	-	-	-	-	-	-	-	-	-	-	-	-	-	-	-	-	-	-	-	-	-	-	-
**Previous degree or qualification**	-	-	-	-	-	-	-	-	-	-	-	-	-	-	-	-	-	-	-	-	-	-	0.213††
No	79	83.16	-	-	8	10.13	-	-	12	15.19	-	-	25	31.65	-	-	34	43.04	-	-	-	-	-
Yes	16	16.84	-	-	2	12.50	-	-	5	31.25	-	-	6	37.50	-	-	3	18.75	-	-	-	-	-
**Medical training institution**	-	-	-	-	-	-	-	-	-	-	-	-	-	-	-	-	-	-	-	-	71.30	33	< 0.001**
University of Pretoria	37	38.95	-	-	2	5.41	-	-	4	10.81	-	-	9	24.32	-	-	22	59.46	-	-	-	-	-
Sefako Makgatho Health Sciences University	15	15.79	-	-	2	13.33	-	-	1	6.67	-	-	6	40.00	-	-	6	40.00	-	-	-	-	-
University of Cape Town	7	7.37	-	-	0	0.00	-	-	3	42.86	-	-	0	0.00	-	-	4	57.14	-	-	-	-	-
University of the Witwatersrand	8	8.42	-	-	0	0.00	-	-	3	37.50	-	-	4	50.00	-	-	1	12.50	-	-	-	-	-
University of the Free State	8	8.42	-	-	0	0.00	-	-	4	50.00	-	-	4	50.00	-	-	0	0.00	-	-	-	-	-
University of KwaZulu-Natal	3	3.16	-	-	0	0.00	-	-	2	66.67	-	-	0	0.00	-	-	1	33.33	-	-	-	-	-
Stellenbosch University	2	2.11	-	-	0	0.00	-	-	0	0.00	-	-	1	50.00	-	-	1	50.00	-	-	-	-	-
University of Limpopo	1	1.05	-	-	0	0.00	-	-	0	0.00	-	-	0	0.00	-	-	1	100.00	-	-	-	-	-
University in Cuba	8	8.42	-	-	2	25.00	-	-	0	0.00	-	-	5	62.50	-	-	1	12.50	-	-	-	-	-
University in China	4	4.21	-	-	3	75.00	-	-	0	0.00	-	-	1	25.00	-	-	0	0.00	-	-	-	-	-
Internationally trained: Other	1	1.05	-	-	1	100.00	-	-	0	0.00	-	-	0	0.00	-	-	0	0.00	-	-	-	-	-
Other	1	1.05	-	-	0	0.00	-	-	0	0.00	-	-	1	100.00	-	-	0	0.00	-	-	-	-	-
**Institutional preparedness**	-	-	-	-	-	-	-	-	-	-	-	-	-	-	-	-	-	-	-	-	2.53	3	0.469
Felt unprepared	49	51.58	-	-	3	6.12	-	-	8	16.33	-	-	17	34.69	-	-	21	42.86	-	-	-	-	-
Felt adequately prepared	46	48.42	-	-	7	15.22	-	-	9	19.57	-	-	14	30.43	-	-	16	34.78	-	-	-	-	-
**Work-related**	-	-	-	-	-	-	-	-	-	-	-	-	-	-	-	-	-	-	-	-	-	-	-
**Internship year**	-	-	-	-	-	-	-	-	-	-	-	-	-	-	-	-	-	-	-	-	0.48	3	0.924
First year	44	46.32	-	-	4	9.09	-	-	7	15.91	-	-	15	34.09	-	-	18	40.91	-	-	-	-	-
Second year	51	53.68	-	-	6	11.76	-	-	10	19.61	-	-	16	31.37	-	-	19	37.25	-	-	-	-	-
**Current departmental rotation**	-	-	-	-	-	-	-	-	-	-	-	-	-	-	-	-	-	-	-	-	25.67	21	0.219
Family medicine	25	26.32	-	-	2	8.00	-	-	2	8.00	-	-	7	28.00	-	-	14	56.00	-	-	-	-	-
Internal medicine	14	14.74	-	-	2	14.29	-	-	1	7.14	-	-	5	35.71	-	-	6	42.86	-	-	-	-	-
General surgery	11	11.58	-	-	0	0.00	-	-	0	0.00	-	-	4	36.36	-	-	7	63.64	-	-	-	-	-
Paediatrics	10	10.53	-	-	1	10.00	-	-	4	40.00	-	-	2	20.00	-	-	3	30.00	-	-	-	-	-
Psychiatry	10	10.53	-	-	2	20.00	-	-	3	30.00	-	-	3	30.00	-	-	2	20.00	-	-	-	-	-
Obstetrics and gynaecology	9	9.47	-	-	1	11.11	-	-	2	22.22	-	-	4	44.44	-	-	2	22.22	-	-	-	-	-
Orthopaedics	9	9.47	-	-	0	0.00	-	-	2	22.22	-	-	4	44.44	-	-	3	33.33	-	-	-	-	-
Anaesthesiology	7	7.37	-	-	2	28.57	-	-	3	42.86	-	-	2	28.57	-	-	0	0.00	-	-	-	-	-
**Overtime hours per month**	-	-	-	-	-	-	-	-	-	-	-	-	-	-	-	-	-	-	-	-	-	-	0.121††
≤ 40 hours	4	4.21	-	-	1	25.00	-	-	0	0.00	-	-	3	75.00	-	-	0	0.00	-	-	-	-	-
> 40 but < 80 hours	55	57.89	-	-	7	12.73	-	-	12	21.82	-	-	18	32.73	-	-	18	32.73	-	-	-	-	-
≥ 80 hours	36	37.89	-	-	2	5.56	-	-	5	13.89	-	-	10	27.78	-	-	19	52.78	-	-	-	-	-
**History of diagnosed mental illness**	-	-	-	-	-	-	-	-	-	-	-	-	-	-	-	-	-	-	-	-	3.47	3	0.324
No	63	66.32	-	-	9	14.29	-	-	12	19.05	-	-	20	31.75	-	-	22	34.92	-	-	-	-	-
Yes	32	33.68	-	-	1	3.13	-	-	5	15.63	-	-	11	34.38	-	-	15	46.88	-	-	-	-	-
**Diagnosed mental illness**	-	-	-	-	-	-	-	-	-	-	-	-	-	-	-	-	-	-	-	-	-	-	-
Major depressive disorder (MDD)	18	56.25	-	-	1	5.56	-	-	3	16.67	-	-	7	38.89	-	-	7	38.89	-	-	-	-	0.790††
Anxiety disorder (AD)	14	43.75	-	-	0	0.00	-	-	3	21.43	-	-	4	28.57	-	-	7	50.00	-	-	-	-	0.790††
Bipolar disorder (BD)	2	6.25	-	-	0	0.00	-	-	0	0.00	-	-	0	0.00	-	-	2	100.00	-	-	-	-	0.667††
Schizophrenia	0	0.00	-	-	0	0.00	-	-	0	0.00	-	-	0	0.00	-	-	0	0.00	-	-	-	-	-
Personality disorder (PD)	1	3.13	-	-	0	0.00	-	-	0	0.00	-	-	1	100.00	-	-	0	0.00	-	-	-	-	0.531††
Obsessive compulsive disorder (OCD)	2	6.25	-	-	0	0.00	-	-	0	0.00	-	-	1	50.00	-	-	1	50.00	-	-	-	-	1.00††
Other- not specified	3	9.38	-	-	0	0.00	-	-	1	33.33	-	-	1	33.33	-	-	1	33.33	-	-	-	-	0.767††
**Co-occurring diagnoses**	-	-	-	-	-	-	-	-	-	-	-	-	-	-	-	-	-	-	-	-	-	-	-
MDD and AD	6	18.75	-	-	-	-	-	-	-	-	-	-	-	-	-	-	-	-	-	-	-	-	-
MDD and PD	1	3.13	-	-	-	-	-	-	-	-	-	-	-	-	-	-	-	-	-	-	-	-	-
MDD and OCD	1	3.13	-	-	-	-	-	-	-	-	-	-	-	-	-	-	-	-	-	-	-	-	-
AD and BD	1	3.13	-	-	-	-	-	-	-	-	-	-	-	-	-	-	-	-	-	-	-	-	-
**Timing of diagnosis**	-	-	-	-	-	-	-	-	-	-	-	-	-	-	-	-	-	-	-	-	-	-	0.106††
Before internship	25	78.13	-	-	0	0.00	-	-	5	20.00	-	-	7	28.00	-	-	13	52.00	-	-	-	-	-
During internship	7	21.88	-	-	1	14.29	-	-	0	0.00	-	-	4	57.14	-	-	2	28.57	-	-	-	-	-
**Worsening of mental illness during internship**	-	-	-	-	-	-	-	-	-	-	-	-	-	-	-	-	-	-	-	-	-	-	0.171††
Yes	18	56.25	-	-	1	5.56	-	-	2	11.11	-	-	4	22.22	-	-	11	61.11	-	-	-	-	-
No	14	43.75	-	-	0	0.00	-	-	3	21.43	-	-	7	50.00	-	-	4	28.57	-	-	-	-	-
**Support and resources**	-	-	-	-	-	-	-	-	-	-	-	-	-	-	-	-	-	-	-	-	-	-	-
**Awareness of counselling services available at SBAH**	-	-	-	-	-	-	-	-	-	-	-	-	-	-	-	-	-	-	-	-	-	-	0.020*††
Unaware of hospital counselling services	79	83.16	-	-	5	6.33	-	-	13	16.46	-	-	28	35.44	-	-	33	41.77	-	-	-	-	-
Aware of counselling services	16	16.84	-	-	5	31.25	-	-	4	25.00	-	-	3	18.75	-	-	4	25.00	-	-	-	-	-
**Family geographical proximity**	-	-	-	-	-	-	-	-	-	-	-	-	-	-	-	-	-	-	-	-	-	-	0.359††
Pretoria	40	42.11	-	-	6	15.00	-	-	8	20.00	-	-	10	25.00	-	-	16	40.00	-	-	-	-	-
Gauteng	29	30.53	-	-	0	0.00	-	-	5	17.24	-	-	12	41.38	-	-	12	41.38	-	-	-	-	-
Family elsewhere in South Africa	25	26.32	-	-	4	16.00	-	-	4	16.00	-	-	9	36.00	-	-	8	32.00	-	-	-	-	-
Family in another country	1	1.05	-	-	0	0.00	-	-	0	0.00	-	-	0	0.00	-	-	1	100.00	-	-	-	-	-
**Perceived emotional support from family or friends**	-	-	-	-	-	-	-	-	-	-	-	-	-	-	-	-	-	-	-	-	-	-	0.368††
Not receiving emotional support	6	6.32	-	-	0	0.00	-	-	0	0.00	-	-	4	66.67	-	-	2	33.33	-	-	-	-	-
Receiving emotional support	89	93.68	-	-	10	11.24	-	-	17	19.10	-	-	27	30.34	-	-	35	39.33	-	-	-	-	-
**Frequency of emotional support received**	-	-	-	-	-	-	-	-	-	-	-	-	-	-	-	-	-	-	-	-	-	-	0.879 ††
Daily	40	44.94	-	-	5	12.50	-	-	9	22.50	-	-	10	25.00	-	-	16	40.00	-	-	-	-	-
Weekly	30	33.71	-	-	3	10.00	-	-	6	20.00	-	-	10	33.33	-	-	11	36.67	-	-	-	-	-
Monthly	15	16.85	-	-	1	6.67	-	-	1	6.67	-	-	6	40.00	-	-	7	46.67	-	-	-	-	-
Few times a year	4	4.49	-	-	1	25.00	-	-	1	25.00	-	-	1	25.00	-	-	1	25.00	-	-	-	-	-

SBAH, Steve Biko Academic Hospital; IQR, interquartile range; *n*, number; *χ*^2^, chi-square test; *df*, degrees of freedom.

* , *p* < 0.05;

** , *p* < 0.001.

† , Age range: 23–45 years;

†† , Fisher’s exact test.

### Empathy

The median JSE score was 106.00 (IQR: 96.00–115.00), with scores ranging from 50.00 to 134.00.

### Factors associated with empathy

Empathy was not significantly associated with the presence of a prior mental illness diagnosis (*χ*^2^ (1) = 0.61, *p* = 0.436). However, worsening of pre-existing mental illness during internship was significantly associated with empathy scores. Interns who reported worsening mental illness had higher median empathy scores (111.50) than those without worsening symptoms (98.50) (*χ*^2^(1) = 5.74, *p* = 0.017).

Age was not significantly correlated with empathy (Spearman’s *ρ* = 0.15, *p* = 0.165).

No significant associations were observed between empathy scores and gender, relationship status, prior degree, medical school attended, perceived institutional preparation, internship year, rotation, overtime hours, awareness of counselling services or social support variables (*p* > 0.05).

### Association between burnout and empathy

Spearman’s rank correlation coefficients demonstrated significant associations between burnout dimensions and empathy scores. Emotional exhaustion (*ρ* = –0.24, *p* = 0.017) and depersonalisation (*ρ* = –0.36, *p* < 0.001) were negatively correlated with empathy, while personal accomplishment was positively correlated with empathy (*ρ* = 0.29, *p* = 0.005).

Empathy scores differed significantly across burnout profiles (*χ*^2^ (3) = 14.86, *p* = 0.002). Interns in the engaged profile had the highest median JSE score (120.50). In contrast, those in the ineffective, overextended and burnout profiles demonstrated lower median scores (103.00, 103.00 and 103.00, respectively). The engaged group also demonstrated less variability in empathy levels. Full results are shown in Table 5.

**TABLE 5:** Maslach Burnout Inventory profile and Jefferson Scale of Empathy scores.

Profile	*n*	%	Median	25th Percentile	75th Percentile	Minimum	Maximum
1. Engaged	10	10.53	120.50	115.00	127.00	109.00	133.00
2. Ineffective	17	17.89	103.00	94.00	109.00	54.00	130.00
3. Overextended	31	32.63	103.00	96.00	120.00	50.00	134.00
4. Burnout	37	38.95	103.00	90.00	112.00	64.00	124.00

Note: Kruskal-Wallis test: *χ*^2^ (3) = 14.86, *p* = 0.002.

A simple linear regression analysis demonstrated that burnout profile significantly predicted empathy scores (*F*[3, 91] = 4.69, *p* = 0.004), explaining 13.4% of the variance in JSE scores (*R*^2^ = 0.134). In a multivariable linear regression model adjusting for age, gender, medical school, worsening of pre-existing mental illness, awareness of counselling services and overtime hours, the overall model remained statistically significant (*F*[10, 84] = 2.56, *p* = 0.009), explaining 23.30% of the variance in empathy scores (*R*^2^ = 0.233). Burnout profile (*p* = 0.004) and worsening pre-existing mental illness (*p* = 0.019) remained significant independent predictors of empathy, while the other variables were not statistically significant.

In additional multivariable analyses examining burnout subscale scores, PA remained a significant positive predictor of empathy (*p* = 0.035), while DP showed a marginal negative association (*p* = 0.088). Emotional exhaustion was not independently associated with empathy. In analyses using categorised burnout subscale severity levels, DP emerged as a significant independent predictor of empathy (*p* = 0.002).

## Discussion

This study investigated the association between burnout and empathy among intern doctors at a tertiary academic hospital. A high prevalence of burnout was observed, with more than 70% of interns classified within the overextended or burnout profiles. Burnout was significantly associated with age, medical school attended and awareness of counselling services. Empathy differed significantly across burnout profiles, with interns in the engaged category demonstrating higher empathy scores than those in the ineffective, overextended and burnout profiles. Additionally, worsening of a pre-existing mental illness during internship was significantly associated with higher empathy scores.

The high prevalence of burnout in this cohort mirrors broader South African data on junior doctors and likely reflects the cumulative effects of prolonged workload, emotional strain and limited institutional safeguards.^6,21^ Although burnout is multifactorial, the observed patterns suggest specific points of vulnerability.^3,6,7,21^

Younger interns were more likely to fall within higher burnout profiles, echoing prior evidence that limited clinical experience and underdeveloped coping strategies may amplify stress responses during early career transitions.^6,21^

An additional association emerged between burnout and the institution from which interns graduated, possibly reflecting differences in preparation for stress management and contextual factors such as proximity to family responsibilities.^22,23,24^ Many interns from nearby universities, particularly those with training sites at or close to the study hospital, may have opted to remain in the area because of familiarity or convenience. While this can be beneficial, it may also introduce additional personal responsibilities outside of work, such as caregiving or family obligations, potentially heightening role strain and emotional fatigue.^22,23,24,25^ In this way, geographic continuity may serve as both a protective and risk factor, depending on the intern’s broader psychosocial context.^22,23,24,25^

Lack of awareness of institutional counselling services was significantly associated with burnout. Interns who were unaware of available support were more likely to fall into the overextended or burnout categories, underscoring the importance of providing mental health services and ensuring that they are clearly communicated, approachable and embedded within institutional systems. This finding aligns with existing literature showing that limited awareness or access to occupational health services can compound psychological strain.^6,26^ Improving awareness and accessibility may be critical in promoting emotional resilience and mitigating burnout.

Other observed patterns did not reach statistical significance but warrant cautious consideration. Interns with a history of mental illness showed a trend towards higher burnout, possibly reflecting greater psychological vulnerability under sustained pressure.

Similarly, higher proportions of burnout were observed among interns rotating through general surgery, family medicine and internal medicine, as well as among those reporting longer working hours. Although these associations were not statistically significant, they are consistent with the literature linking chronic workload strain to fatigue and professional disengagement.^3,6,7^

Overall, these results highlight that burnout arises from individual vulnerability and systemic, institutional and interpersonal pressures. Addressing this requires structural change, including manageable workloads, accessible support systems and a culture prioritising internal well-being as fundamental to safe and compassionate care.

Interestingly, interns who reported worsening mental health during internship had higher empathy scores, suggesting that heightened emotional sensitivity may be associated with increased psychological vulnerability. This reflects an ‘empathy paradox’, in which the same responsiveness that benefits patient care may also render clinicians more susceptible to distress in demanding clinical settings.^13^ The persistence of this association in the adjusted model further suggests that worsening of a pre-existing mental illness may be independently associated with higher empathy scores in this cohort.

Although most demographic and institutional factors were not significantly associated with empathy, five non-significant trends were observed. Interns with a history of mental illness tended to report higher empathy. In comparison, lower empathy was observed among those lacking emotional support, those unaware of available counselling services and those rotating through high-demand departments such as general surgery and obstetrics and gynaecology. Lower empathy was also observed among interns reporting longer overtime working hours. As these associations did not reach statistical significance, they should be interpreted cautiously. However, they align with previous literature suggesting that inadequate support systems, heavy workloads and demanding clinical environments may be associated with reduced empathic capacity.^1,14^

This study identified a significant inverse association between burnout and empathy among intern doctors. Engaged interns reported higher empathy, whereas those with higher burnout demonstrated reduced empathy. Importantly, this association remained significant after adjustment for demographic, clinical and work-related variables, suggesting that the relationship between burnout and empathy was not solely explained by background characteristics or work context. While previous literature has often highlighted EE as the burnout component most closely linked to reduced empathic engagement, our findings suggest a more nuanced pattern.^13^ Although higher EE and DP were both associated with lower empathy in the correlation analyses, exploratory multivariable analyses indicated that PA remained positively associated with empathy, whereas DP showed a marginal negative association.

When categorised burnout subscale levels were examined, DP severity emerged as a significant independent predictor of empathy. This may help explain the growing emphasis on burnout profiles rather than isolated subscales, as profile-based approaches may better reflect the multidimensional nature of burnout and may therefore offer a more useful way of examining its relationship with empathy.^19^

A key strength of the study was the use of an internationally validated, gold-standard measure of burnout and a validated, widely used measure of empathy among healthcare professionals and students.^18,27^ The study achieved a strong response and completion rate, with participants from all departments and internship years, thus improving representativeness. Appropriate psychological support services were available to participants throughout the study period.

The findings highlighted in this study should be interpreted in light of important limitations. The single-site, cross-sectional design limits generalisability and prevents causal inference. Self-reporting raises the possibility of social desirability bias or under-reporting. Key confounding variables, such as coping styles and personality traits, were not assessed. Data collection occurred during a high-stress period (December to February), when staffing levels are usually reduced because of many personnel being on leave. The decreased workforce, combined with an unchanged patient load, may have contributed to higher burnout scores during this period. Some subgroups had too few participants to support robust statistical comparisons. Qualitative data were not collected, limiting insights into interns’ lived experiences.

### Recommendations

The findings highlight educational and systemic gaps. Undergraduate medical curricula should integrate stress management and emotional resilience training to prepare interns for the psychological demands of clinical work. Hospitals should improve awareness and accessibility of counselling services through enhanced orientation and efforts to destigmatise mental health care. Structural changes should aim to reduce excessive work hours and increase psychological support. Future research should adopt longitudinal and qualitative approaches and include multi-centre studies to improve generalisability and explore the development of burnout and empathy over time.

## Conclusion

This study revealed a high prevalence of burnout among South African medical interns at SBAH, with more than 70% of interns classified within the overextended or burnout profiles and only a small proportion falling within the engaged profile. Empathy was significantly lower among interns with higher burnout, and this inverse association remained significant after adjustment for demographic, clinical and work-related factors.

This has implications for both clinician well-being and patient care. Worsening of a pre-existing mental illness during internship was also independently associated with higher empathy scores.

Younger age, medical school attended and lack of awareness of counselling services were significantly associated with burnout. These findings suggest that both individual and institutional factors contribute to burnout risk during internship. Addressing these systemic, institutional and educational factors should be considered a healthcare priority to protect both medical trainees and the patients they serve.
